# Deciphering the genetic and epidemiological landscape of mitochondrial DNA abundance

**DOI:** 10.1007/s00439-020-02249-w

**Published:** 2020-12-31

**Authors:** Sara Hägg, Juulia Jylhävä, Yunzhang Wang, Kamila Czene, Felix Grassmann

**Affiliations:** 1 Department of Medical Epidemiology and Biostatistics, Karolinska Institutet, Nobels väg 12A, 171 65 Stockholm, Sweden; 2 Institute of Medical Sciences, University of Aberdeen, Aberdeen, UK

## Abstract

Mitochondrial (MT) dysfunction is a hallmark of aging and has been associated with most aging-related diseases as well as immunological processes. However, little is known about aging, lifestyle and genetic factors influencing mitochondrial DNA (mtDNA) abundance. In this study, mtDNA abundance was estimated from the weighted intensities of probes mapping to the MT genome in 295,150 participants from the UK Biobank. We found that the abundance of mtDNA was significantly elevated in women compared to men, was negatively correlated with advanced age, higher smoking exposure, greater body-mass index, higher frailty index as well as elevated red and white blood cell count and lower mortality. In addition, several biochemistry markers in blood-related to cholesterol metabolism, ion homeostasis and kidney function were found to be significantly associated with mtDNA abundance. By performing a genome-wide association study, we identified 50 independent regions genome-wide significantly associated with mtDNA abundance which harbour multiple genes involved in the immune system, cancer as well as mitochondrial function. Using mixed effects models, we estimated the SNP-heritability of mtDNA abundance to be around 8%. To investigate the consequence of altered mtDNA abundance, we performed a phenome-wide association study and found that mtDNA abundance is involved in risk for leukaemia, hematologic diseases as well as hypertension. Thus, estimating mtDNA abundance from genotyping arrays has the potential to provide novel insights into age- and disease-relevant processes, particularly those related to immunity and established mitochondrial functions.

**Supplementary Information:**

The online version contains supplementary material available at 10.1007/s00439-020-02249-w.

## Introduction

Mitochondria (MT), in addition to producing energy through oxidative processes, are involved in heat production, ion storage, apoptosis, intra- and extra-cellular cell signalling, biosynthesis and degradation of important metabolites as well as the processing of therapeutic agents. Depending on the tissue, cells host a dynamic range of multiple mitochondria which in turn contain multiple copies of a tiny circular genome (16,569 base pairs). The human mitochondrial proteome consists of more than 1100 proteins, of which only 13 are encoded in mitochondrial genome (Calvo and Mootha 2010). In contemporary human populations, several haplogroups are defined by ancestral and stable mutations in the MT genome. Those haplogroups are derived from adaptation to different geographic areas under distinctive selection pressure (Torroni et al. 1996) and may also influence the abundance of mitochondrial DNA (mtDNA) (Liou et al. 2010; Gómez-Durán et al. 2012), although there is conflicting evidence (Hulgan et al. 2019; Pyle et al. 2016). Notably, different haplogroups have been reported to be associated with diseases as well as longevity (Benedictis et al. 1999; Chinnery and Gomez-Duran 2018) and as such contribute to phenotypic variety within human populations. Mitochondria rely on self-replication and are thus prone to cellular stress in aging and diseases. Hence, MT dysfunction and a reduction in MT biogenesis are hallmarks of aging (López-Otín et al. (2013)), and have been associated with most aging-related diseases (Haas 2019) as well as immunological processes (McGuire 2019).

Indeed, prior reports indicate that the amount of mtDNA in the blood decreases with advanced age (McArdle et al. 2013; Moore et al. 2018) as well as with lifestyle factors such as increased smoking behaviour (Cardellach et al. 2003) and higher BMI (McArdle et al. 2013). In addition, males were reported to have a lower amount of mtDNA in their blood compared to women (Knez et al. 2016), which may be due to differing levels of white blood cells (Shim et al. 2020) or oestrogen levels (Giordano et al. 2011) between the sexes. However, it remains unclear whether the changes in mtDNA abundance also related to a reduced number of mitochondria in the respective cell population or due to reduced genome copies within the mitochondria.

To our knowledge, there is presently no comprehensive evaluation of aging or other factors influencing mtDNA abundance in nucleated blood cells in a large collection of individuals. In addition, few studies have been conducted to elucidate the inherited genetic contribution to mtDNA abundance (Workalemahu et al. 2017; Guyatt et al. 2019; López et al. 2014; Cai et al. 2015; Curran et al. 2007), most of which include a low number of individuals. Therefore, we aimed to leverage data from the UK Biobank, a cohort of more than 500,000 participants aged 40 and above, to uncover non-modifiable as well as lifestyle factors associated with mtDNA abundance and to further establish a role of inherited genetics in shaping the abundance of MT.

## Results

We estimated the amount/abundance of mitochondrial DNA in blood in 295,150 individuals passing quality control (Supplementary Fig. 1 and Table 1) from the weighted intensities of genotyping probes mapped to the MT genome (Supplementary Table S1). We noticed that the individual genotyping batches (each containing around 5000 individuals) were contributing to the variance of the estimated mtDNA abundance (Supplementary Fig. 1). In addition, we found a strong effect of genotyping missingness [slope per SD and 95% confidence intervals (CI) 0.011 (0.008; 0.015), *P* < 10^–10^] on the abundance of mitochondrial DNA in UK Biobank participants. Hence, we adjusted our analyses by both the genotyping batch as well as genotyping missingness.

**Table 1:** Baseline characteristics of UK Biobank participants by sex

Variable	UK biobank participants		
	Females	Males	Both
Number of individuals	158,762	136,388	295,150
Average age (SD) [years]	56.67 (7.90)	57.14 (8.09)	56.89 (7.99)
Average body mass index (SD) [kg/m^2^]	27.03 (5.12)	27.83 (4.21)	27.40 (4.74)
Average packyears smoked (SD)	6.40 (12.89)	11.05 (18.85)	8.52 (16.05)
Average mtDNA abundance (SD)^a^	0.02 (0.99)	− 0.03 (0.99)	0.00 (0.99)
Median frailty index (IQR)^b^	0.11 (0.01–0.21)	0.11 (0.01–0.20)	0.11 (0.01–0.21)
Median call rate (range)	1.00 (0.99–1.00)	1.00 (0.99–1.00)	1.00 (0.99–1.00)

*SD* standard deviation, *IQR* interquartile range

^a^Average mtDNA abundance (mL2RMT) estimated from the weighted genotyping probe intensities (L2R), scaled to have a mean of zero and a SD of 1.00 in each genotyping plate

^b^Median fraction of reported health related deficits out of 49 investigated deficits

### Association of mitochondrial DNA abundance with sex, aging and lifestyle factors

Previous studies implicated a strong role of sex and advanced age in mtDNA abundance. Indeed, we found that male sex [slope and 95% CI − 0.05 (− 0.057; − 0.043); *P* < 10^–43^] and advanced age (slope per SD and 95% CI − 0.06 (− 0.064; − 0.056); *P* < 10^–238^] was strongly associated with mtDNA abundance. Since mtDNA abundance may depend on oestrogen levels and thus may be influenced by menopausal status in women, we next investigated whether menopausal status influences mtDNA abundance changes with advanced age. To this end, we used a two-lines test to programmatically determine the optimal age cut-off, which found that the association of age with mtDNA abundance has differential effects in women before and after 55 years of age (Supplementary Fig. 2). We found that mtDNA abundance increases with age in pre-menopausal women and decreases in post-menopausal women. In contrast, mtDNA abundance decreases steadily in men with advanced age and no differences between age groups was observed.

**Fig. 1:**
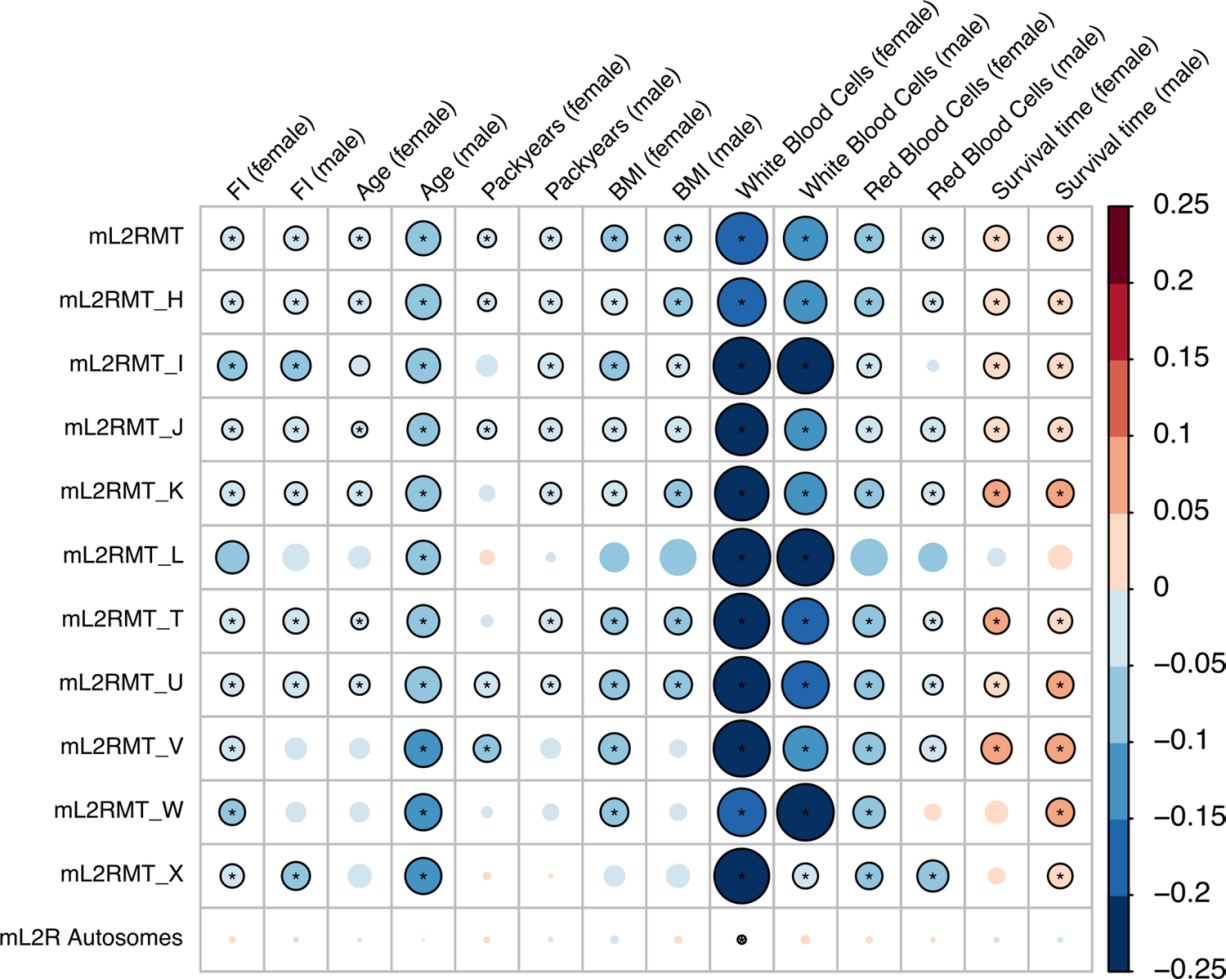
Association between mitochondrial DNA abundance and different covariates. The size and colour (see colour bar) of the circle represent the slope of the association of seven features with mitochondrial (mL2RMT) as well as autosomal DNA abundance (mL2R Autosomes), stratified by sex (for the precise effect sizes see also Supplementary Table S2). The associations were also analysed separately by major MT haplogroup (labelled H–X). Correlations which were statistically significant (*Q* value < 0.05) are indicated with an asterisk. Nominally significant associations (*P* value < 0.05) are shown as circles with black borders. *FI* frailty index, *BMI* body mass index, *mL2RMT* median log 2 ratio of mitochondrial probes; survival time, time till death or end of follow-up

Due to the strong effect of sex on mtDNA abundance as well as the differential effect of advanced age on mtDNA abundance in pre- and post-menopausal women, we present all subsequent results separately for males and females. In addition to sex and increasing age, we also found that more packyears and elevated BMI were negatively associated with MT DNA abundance in both men and women (*Q* value < 0.05, Fig. 1, Supplementary Table S2). In addition to those results, we found that mtDNA abundance is negatively associated with the increased frailty as expressed by the frailty index, an accumulation deficit model summarizing self-reported symptoms and diagnoses (*Q* value < 0.05, Fig. 1, Supplementary Table S2). In line with those results, individuals with higher mtDNA abundance had a statistically significant better/longer survival time after recruitment.

While we found that the abundance of mtDNA was related to the haplogroup of the individuals (Supplementary Figure S3), similar and consistent effect sizes were observed for the most common haplogroups present in the UKB, indicating that the genetic make-up of the MT itself is not a major factor in determining mtDNA abundance changes due to age or other factors (Fig. 1, Supplementary Table S2).

### Mitochondrial abundance and blood markers

**Fig. 2:**
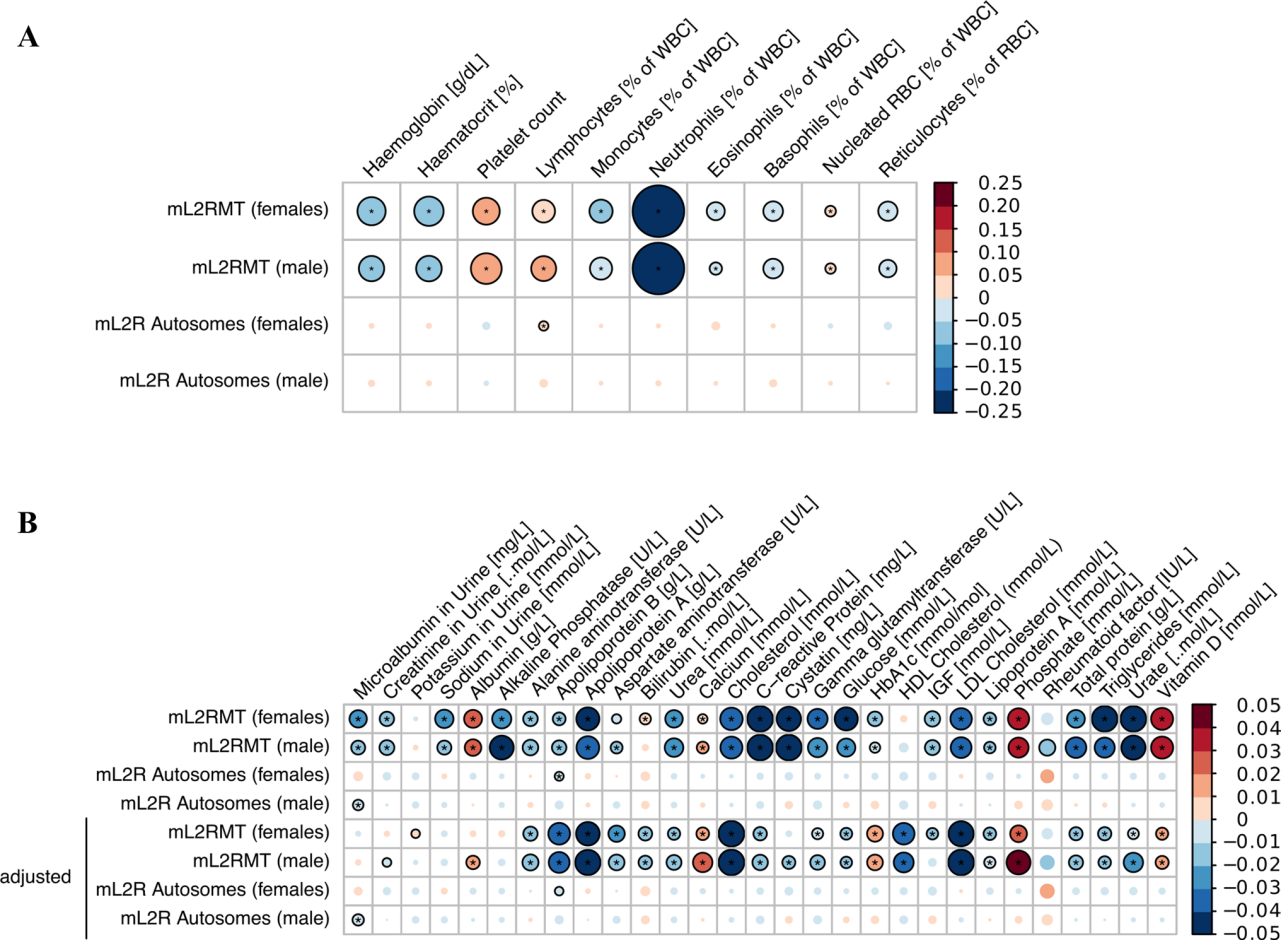
Association between mitochondrial DNA abundance and individual blood markers. The size and colour of the circle represent the slope of the correlation of blood cell counts or other blood measurements (**a** see also Supplementary Table S3) and biochemistry features (**b** see also Supplementary Table S4) with mitochondrial (mL2RMT) as well as autosomal DNA abundance (mL2R Autosomes), stratified by sex. In **b** the association of biochemistry markers with mtDNA abundance were additionally adjusted for the neutrophil and lymphocyte percentage (% of white blood cells) as well as white blood cell count in the last four rows. Correlations which were statistically significant (*Q* value < 0.05) are indicated with an asterisk. Nominally significant associations (*P* value < 0.05) are shown as circles with black borders. *WBC* white blood cells, *RBC* red blood cells, *mL2RMT* median log 2 ratio of mitochondrial probes, *NRBC* nucleated red blood cells

Next, we investigated whether the amount of DNA that is hybridized to the genotyping arrays may be directly related to the number of nucleated cells per volume of blood. Indeed, we found that the white blood cell count was negatively associated with mtDNA abundance (Fig. 1, Supplementary Table S2). However, we also found that increased red blood cell count was associated with reduced mtDNA abundance, indicating that not only the number of nucleated cells but also other mechanisms seem to play a role in governing mtDNA abundance. This conclusion is supported by the finding that we only observed a weak positive correlation of the aggregated autosomal DNA abundance with white blood cell count, which was estimated from all genotyping probes mapped to autosomes (Fig. 1). We also investigated individual blood markers and their association with mtDNA abundance and found that the mtDNA abundance was negatively linked to neutrophil, eosinophil, basophil and monocyte percentage and positively linked to lymphocyte percentage and platelet count (Fig. 2a, Supplementary Table S3). In contrast, autosomal DNA abundance was only significantly positively associated with lymphocyte percentage in females.

### Mitochondrial abundance and biochemistry markers

When we investigated the association of 29 biochemistry markers (from urine and blood, Fig. 2b, Supplementary Table S4) and mtDNA abundance, we noticed significant associations with markers related to inflammation (C-reactive protein), kidney function (albumin, cystatin c, urea and urate as well as sodium in urine), liver function (alkaline phosphatase, alanine aminotransferase), cholesterol metabolism (LDL cholesterol, total cholesterol, triglycerides, apolipoprotein A) as well as ion homeostasis (calcium and phosphate), vitamin D levels and glucose metabolisms (IGF, glucose, HbA1c). Since the immune system and thus the number of immune cells may well influence the observed associations, we also present the analyses adjusted for the neutrophil and lymphocyte percentage as well as white blood cell count measured in each participant (Fig. 2b, Supplementary Table S4). After adjustment, most of the associated markers remained statistically significantly associated with mtDNA abundance, albeit with lower effect sizes. Of note, markers related to cholesterol metabolisms such as LDL and HDL cholesterol, apolipoprotein A and B as well as total cholesterol had even greater effect sizes after adjustment for immune cell count. In contrast, we found few associations of biochemistry markers with autosomal DNA abundance in our analyses. In particular, the only microalbumin in urine was found to be negatively associated to autosomal DNA abundance in men (*Q* value < 0.05).

### Genetic dissection of mitochondrial abundance

**Fig. 3:**
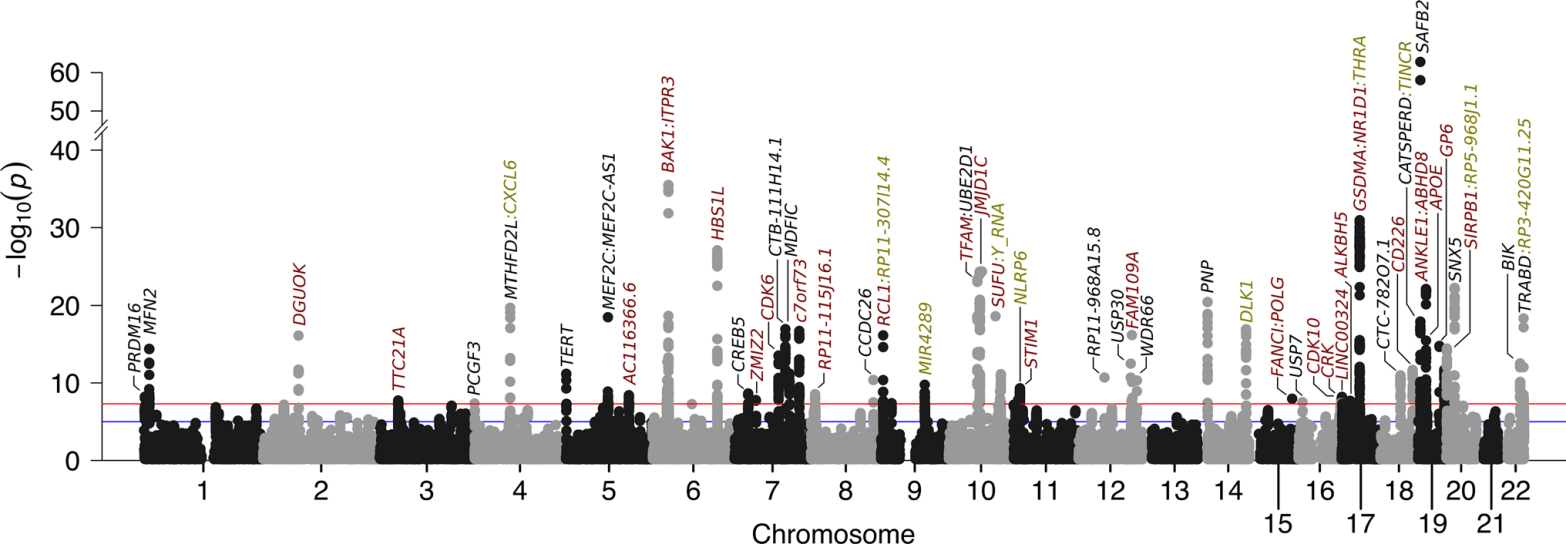
Summary of the genome-wide association study of mtDNA abundance. Manhattan plot of 3,505,788 variants with minor allele frequency greater 5%. Genes influenced by associated variants are highlighted in different colours: genes in red harbour exonic variants, genes which have associated variants in the intronic region are shown in black. In case no intronic or exonic variants are mapped to genes in a region, we highlighted the closest gene in green. The red line denotes genome-wide significance (*P* < 5.00 × 10^–08^) while the blue line denotes suggestive evidence for association (*P* < 1.00 × 10^–05^). The estimated genomic inflation factor *λ* was 1.147

To uncover genetic variants associated with mitochondrial abundance, we conducted a genome-wide association study (GWAS) in all individuals passing quality control. In total, we assessed the association of 3,505,788 variants with mtDNA abundance and found 50 regions in the genome with genome-wide significant variants (*P* value < 5 × 10^–08^, Fig. 3). In those regions, a total of 66 independent signals were detected at genome-wide significance (Supplementary Table S5), implicating multiple genes to be involved in mtDNA abundance (Fig. 3). Importantly, by positional mapping of likely functional variants, we identified genes related to the immune system (*CXCL6, MEF2C, ITPR3, UBE2D1, C7orf73, STIM1, PNP, CRK* and *SIRPB1*), cancer and cell cycle (*TERT, BAK1, CDK6, CDK10, SUFU, FANCI, MDFIC, JMJD1C, USP7, BIK*) and mitochondrial function (*MFN2, TFAM, DGUOK, USP30, CREB5, POLG*) to be potentially involved in governing mtDNA abundance in blood (Fig. 3).

Next, we used fastBAT to investigate the burden of mtDNA abundance associated variants within the gene bodies of all protein-coding genes. In total, 227 genes carrying a statistically significant burden were identified (*P* value < 2 × 10^–06^, Supplementary Table S6). We then investigated a potential overrepresentation of pathways within those genes with WebGestaltR. With this approach, an overrepresentation of pathways related to immune activation, cell–cell adhesion, haematopoiesis, apoptosis, platelet production as well as mitochondrial biogenesis and plasma lipoprotein assembly was observed (Supplementary Figure S4 and Supplementary Table S7).

Finally, we used mixed-effects models to estimate the heritability of mtDNA abundance that can be explained by the genotyped variants. Accounting for age, sex, genotyping missingness, genotyping batch, the first ten genotyping PCs, neutrophil and lymphocyte percentage as well as white blood cell count, we estimated the SNP-heritability to be 8.3% (95% CI 7.6–9.0%), strongly implicating a role of inherited genetic variants on mtDNA abundance.

### PheWAS of mitochondrial abundance

**Fig. 4:**
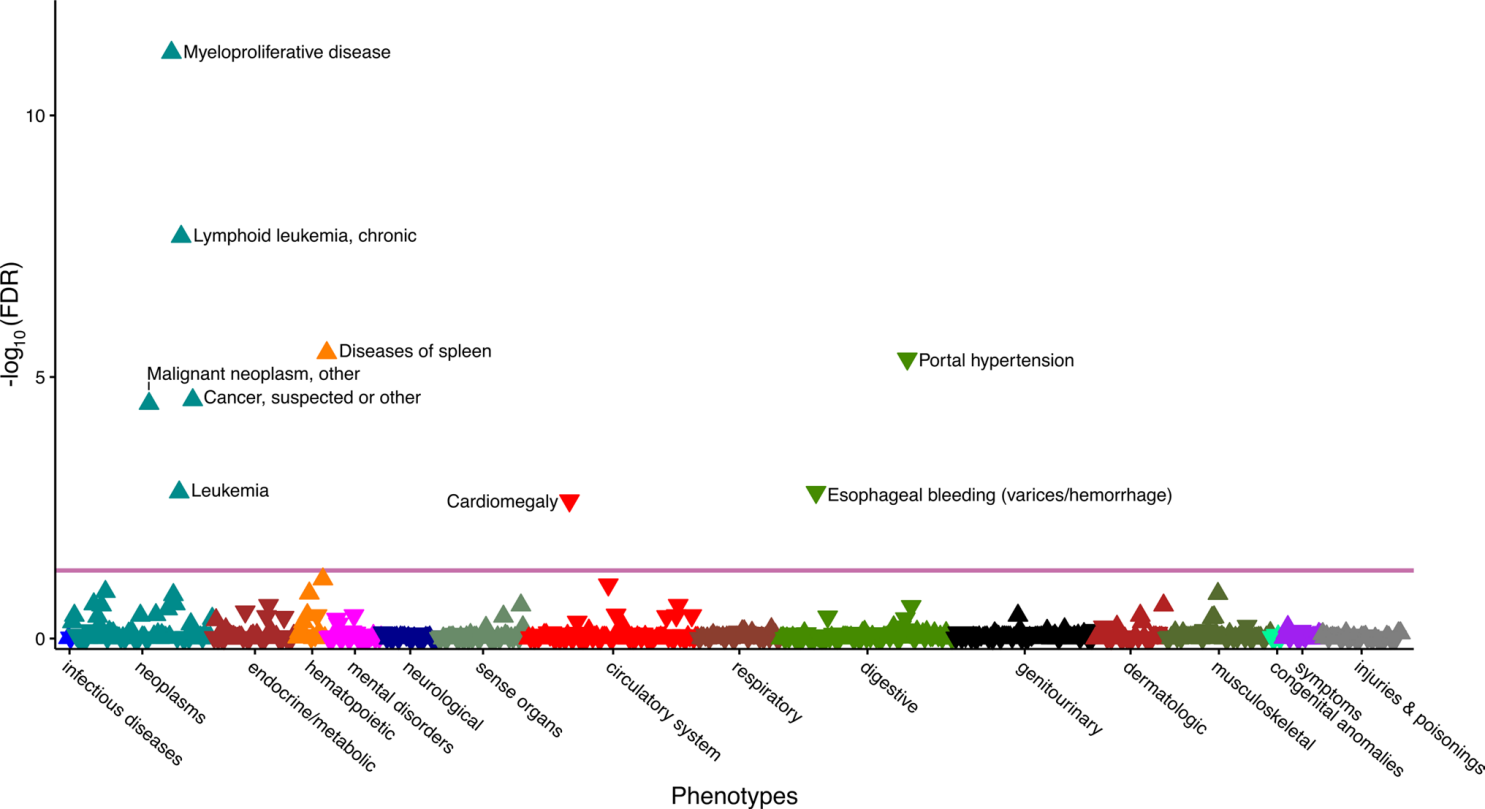
Phenome-wide association study of mtDNA abundance. The consequence of altered mtDNA abundance on 1150 incident diseases or traits determined from the hospital episode spell data was evaluated in all UK Biobank participants (detailed association results are presented in Supplementary Table S8). ICD10 diagnoses were mapped to phecodes and the association between discretized mtDNA abundance (in tertiles) and the phecodes was estimated with logistic regression, adjusted for age at baseline, genotyping batch, total autosomal DNA abundance, fraction of neutrophils, fraction of lymphocytes, white blood cell count, sex, BMI and smoking at baseline as well as the first two principal components of ancestry. Only phecodes with more than 250 cases were considered. Statistically significant associations (*Q* value < 0.05) are shown above the red line. Triangles facing up indicate increased risk while triangle pointing down indicate reduced risk to due elevated mtDNA abundance

To estimate the consequence of altered mtDNA abundance on disease risk, we performed a Phenome-wide association study (PheWAS) on ICD10 codes derived from the hospital episode spell data mapped to a total of 1,150 phecodes. We found that higher levels of mtDNA abundance were statistically significantly (*Q* value < 0.05) associated with increased risk for (chronic lymphoid) leukaemia, myeloproliferative disease as well as diseases of the spleen (Fig. 4 and Supplementary Table S8). In contrast, higher mtDNA abundance resulted in reduced incidence of cardiomegaly, portal hypertension as well as oesophageal bleeding (Fig. 4 and Supplementary Table S8). Finally, prior reports indicated that the risk increase due to differences in mtDNA abundance may follow a U-shaped distribution (i.e. first and third tertile compared to the second tertile increased the risk for a disease) (Workalemahu et al. 2016; Thyagarajan et al. 2012). To test this hypothesis, we have repeated the PheWAS to compare the lowest or the highest tertile of the mtDNA abundance distribution against the second (middle) tertile. However, we found that this approach yielded similar results as obtained from modelling mtDNA abundance across all tertiles. In addition, we observed no additional diseases statistically significantly associated with mtDNA abundance after correction for multiple testing.

## Discussion

In this study, we have explored the epidemiological and genetic basis of mtDNA abundance in nucleated blood cells. We found that age as well as multiple lifestyle and health-related factors were associated with the amount of mtDNA in blood. In particular, mtDNA abundance seems to be mostly influenced by cells involved in the innate immune system as well as cholesterol metabolism and ion homeostasis. In addition, our genetic dissection of mtDNA abundance revealed 50 independent GWAS signals as well as estimated the heritability of mtDNA abundance to be around 8%. Finally, we showed that elevated mtDNA abundance is associated with risk for incident leukaemia, hypertensive disorders and oesophageal bleeding.

Due to the low number of variants and thus probes measuring the abundance of the MT genome, we expected that the mtDNA abundance estimates from chip arrays to be noisy. Indeed, we found that our initial (unweighted) mtDNA abundance values were not associated with known mtDNA correlates such as age, smoking, white blood cell count or BMI in a fashion that is consistent with the literature and was also negatively correlated to the coverage of the MT genome by exome sequencing. Since prior studies suggested that normalized exome coverage provides a robust estimate of mtDNA abundance and also has higher power to detect differences (Zhang et al. 2017; Picardi and Pesole 2012; D’Erchia et al. 2015; Longchamps et al. 2020), we, therefore, weighted the L2R values of individual array probes to more closely resemble the mtDNA abundance estimates from exome sequencing. With this approach, we found that both estimates are moderately positively correlated (*R* = 0.33) and that the influence of genotype missingness on the weighted mtDNA abundance estimate was reduced more than tenfold [slope per SD and 95% CI of genotype missingness correlated to the mL2RMT estimate from MoChA: 0.146 (0.143; 0.150), *P* < 10^–250^]. Since the intensity data does not contain any missing values (i.e. the signal intensity gets recorded for all probes), the decreased influence of genotyping missingness is unlikely to be attributed to the selection of probes with more complete genotyping data. Furthermore, the resulting weighted mtDNA abundance estimates were associated with most covariates in a fashion that is in line with prior reports while the unweighted estimates were not (Supplementary Fig. 5). Thus, while the weighted mtDNA abundance estimates may not necessarily be as accurate as those determined from qPCR, we can estimate mtDNA abundance in the whole UKB cohort, drastically increasing our power to detect associations.

Although the effect of genotype missingness on MT abundance estimation is strongly reduced with our approach, it could still potentially confound our analyses. As such, we adjusted our analyses for the call rate to account for those differences. Therefore, it is unlikely that the genotyping missingness after adjustment would strongly confound most of our analyses, particularly since genotyping missingness itself was not associated with any of the investigated markers in this study (*Q* values > 0.05, data not shown). To estimate the SNP-heritability of mtDNA abundance, the BOLT-LMM algorithm computes the relationship between individuals from all genotyped variants passing QC. Unless genotyping missingness is more strongly pronounced in more related individuals, the estimation of relatedness should not be majorly confounded, particularly after adjusting for the actual genotyping missingness. Our results thus strongly implicate that the amount of mitochondrial DNA in nucleated blood cells is heritable. Further genetic studies accounting for missingness, DNA quality as well as immune cell count are warranted to uncover the precise genetic changes responsible for alternations in mtDNA abundance.

The disparities in mtDNA abundance by sex can be attributed to multiple factors which differ between the sexes. In this study, we were particularly interested in the effect of oestrogen exposure since it has been hypothesized to influence mtDNA abundance (Giordano et al. 2011). As such, we have investigated whether menopause status plays a role in determining mtDNA abundance. Indeed, the amount of mtDNA increases in pre-menopausal women with advanced age but decreases in post-menopausal women at the same rate as observed in males. This observation also explains the reduced effect sizes of age observed on the mtDNA abundance in women in the sex-stratified analyses. The programmatically determined age cut-off is in line with prior studies which showed that 95% of women are post-menopausal at age 55 (Nejat and Chervenak 2010) and using 55 years as the cut-off for menopause status should be sufficient in most research settings (Phipps et al. 2010). In addition, our results may also provide an explanation for the inconsistent results observed in the associations of mtDNA abundance and breast cancer (Lemnrau et al. 2015; Shen et al. 2015, 2010; Thyagarajan et al. 2013; Campa et al. 2018), which is a largely hormonally driven cancer with a strong influence of menopause status on the resulting tumour characteristics such as receptor status. Similarly, our results may also have implications for the investigation of other diseases which are influenced by changes in hormone levels and/or menopause status.

Importantly, our results indicate that mtDNA abundance is negatively associated with increased age, higher frailty, increased smoking behaviour as well as increased BMI, in agreement with prior publications. We hypothesized that MT copy number may be diminished due to lower rates of replication with increasing age (McArdle et al. 2013; Moore et al. 2018), due to oxidative stress induced by smoking (Cardellach et al. 2003), by low-grade inflammation and metabolic stress due to higher BMI (McArdle et al. 2013) or due to prior health-related conditions (as expressed by the frailty index) (Ashar et al. 2015; Bennett and Molofsky 2019). Since individuals with a healthier lifestyle tend to have higher mtDNA abundance, we also observed a reduced incidence of hypertensive disorders such as portal hypertension and cardiomegaly as well as lower mortality in those individuals. In our GWAS, genes related to blood pressure (*NLRP6*, *CREB5* and *APOE*) were found in regions associated with mtDNA abundance, providing a potential explanation between increased mtDNA abundance and reduced risk for hypertensive diseases. Similarly, pathways and genes related to coagulation, platelet production, and count were implicated by our GWAS, thus pointing towards genes that are involved in both mtDNA abundance and risk for oesophageal bleeding. Interestingly, recent reports indicated that higher MT abundance is protective against oesophageal cancer (Xu et al. 2013), which may indicate that the oesophageal tissue is particularly vulnerable to changes in mitochondrial DNA abundance.

To further reinforce the notion that mtDNA abundance is heritable, we conducted a genome-wide association study on over 3 million common variants. We chose to focus on common variants since they are less likely to suffer from missing genotypes and the imputation of missing alleles is more accurate for those variants as well. Our GWAS implicated several genes to be involved in governing mtDNA abundance, particularly those related to immunity, cancer, cell cycle, and mitochondrial function. This is in line with reports that the loss of Y chromosome (mLOY) is related to similar pathways (Thompson et al. 2019), although none of the genes implicated in this study overlap with the genes associated with mLOY. Furthermore, we were able to identify variants significantly associated with mtDNA abundance within a region on chromosome 17 (association signal near *GSDMA*, i.e. genomic locus 40 in Supplementary Table S2), which was previously shown to be involved in mtDNA abundance [estimated from quantitative PCR (Guyatt et al. 2019)]. This region was also described to be involved in blood cell counts, particularly neutrophils (Astle et al. 2016; Okada et al. 2010; Lin et al. 2017). Therefore, our approach does seem to accurately capture mtDNA abundance and provides evidence for additional regions in the genome and thus genes involved in mtDNA abundance.

We observed a strong negative association between mtDNA abundance and the number of neutrophils and a positive correlation with lymphocytes percentage. Since neutrophils are the most abundant leukocyte and thus the most abundant cell carrying DNA, it may seem obvious that the amount of DNA hybridized to the array should be positively correlated. However, our results rather point to other conclusions. First, we did not observe any association of autosomal DNA abundance with the neutrophil count, indicating that the total amount of DNA that was hybridized does not play a major role, potentially due to careful harmonization of DNA concentration used in the genotyping process. Second, we observed differential correlation for mtDNA abundance with myeloblast-derived and lymphoid immune cells. Since both immune cell lineages carry mitochondria, they should similarly contribute to increased mtDNA abundance in case the measured mtDNA abundance is solely dependent on DNA content in blood. Therefore, we conclude that other processes must be responsible for the observed association between increased mtDNA abundance and reduced (innate) immune cell count.

In our PheWAS analysis, we found increased mtDNA abundance to be associated with increased risk of leukaemia, which is in agreement with prior findings which found higher mtDNA abundance to cause increased leukaemia risk (Lan et al. 2008; Kim et al. 2015). Since an increased number of circulating lymphocytes is a hallmark of lymphoid leukaemia (Kipps et al. 2017) and we found a positive association of mtDNA abundance and lymphocyte count, this association would seem obvious. However, we adjusted our PheWAS by neutrophil and lymphocyte percentage as well as white blood cell count, thus the observed effect should be largely independent of immune cell abundance. Since platelets also contain mitochondria and their count can increase acutely through various stimuli (Rokkam and Kotagiri 2020), we additionally adjusted the PheWAS analysis by platelet count and found virtually the same results (results not shown). Those analyses implicate that other mitochondrial mechanisms independent of white blood cell count are responsible for the increased risk. Importantly, in our GWAS, which was also adjusted for white blood cell counts, we found association signals near genes involved in leukaemia and lymphoma development (*CRK, BAK1, CDK6, MDFIC, BIK*) as well as a significant enrichment of pathways related to immune cell proliferation. Thus, further functional studies are necessary to identify the underlying mechanism and responsible (immune) cell population that links the function of those genes to mtDNA abundance and leukaemia.

Of note, the mtDNA estimation was performed at recruitment of the patients and we excluded pre-existing conditions. This allowed us to assess the association of mtDNA abundance with diseases in a prospective manner and thus our results are unlikely influenced by prevalent diseases, which have been shown to alter mtDNA abundance (Ide et al. 2001). The clinical utility if assessing mtDNA estimation remains to be evaluated and would face challenges with our approach: Particularly, the observed effect sizes per tertile are in line with a moderate increase in risk largely independent of known factors influencing mtDNA abundance. Thus, the accuracy to predict the occurrence of those diseases would not be great and required additional risk factors in a comprehensive model. Therefore, further work is necessary to assess the clinical utility of mtDNA estimation, particularly while accounting for known risk factors for those particular diseases. Nevertheless, mtDNA will be a useful marker to add to the increasing number of biomarkers reflecting the human aging process, and as such will be interesting to study further (Jylhävä et al. 2017).

Multiple blood and urine bound biochemistry markers related to cholesterol and triglyceride metabolism as well as ion homeostasis and vitamin D levels were found to be associated with mtDNA abundance independent of neutrophil and lymphocyte percentage or white blood cell count. This is in line with prior research (Liu et al. 2005) and the proposed function of mitochondria in cells since they are instrumental in the metabolism of cholesterol and other lipids as well as in ion storage, particularly phosphate and calcium.

A recent study presented evidence that cell-free and respiratory competent mitochondria are present in blood and may have a role in cell–cell communication (Al Amir Dache 2020). Although the majority of DNA used for the genotyping experiments should be derived from nucleated cells, our current approach can, however, not distinguish between cellular and cell-free mitochondria. It is therefore possible that some of the observed associations are additionally driven by extracellular mitochondria, which warrants further investigations.

## Conclusion

We show that the amount of mtDNA in nucleated blood cells is heritable and associated with different modifiable and non-modifiable markers related to aging, health and lifestyle as well as immunity, cholesterol metabolism and ion homeostasis. Our findings suggest that estimating mtDNA abundance from weighted intensities on genotyping arrays is a promising tool to gain novel insights into disease-relevant mechanisms as well as to study aging.

## Methods

### UK Biobank study population and DNA extraction

UK Biobank (UKB) is a multi-centre cohort with 502,621 participants recruited in England, Scotland and Wales. Genotype data were available for 488,279 participants (Sudlow et al. 2015). The UK Biobank collected two 10 ml EDTA vacutainers per participant and extracted 850 µl of buffy coat, which was randomly distributed on extraction plates and stored at − 80 °C. The DNA for all participants was extracted from the buffy coat aliquot with the Promega Maxwell^®^ 16 Blood DNA Purification Kit (AS1010). In this method, DNA is purified using magnetic particles which are used to capture, wash and elute DNA. Several quality control measures were evaluated after DNA purification and only plates that passed this step were used in genotyping (Welsh et al. 2017). In addition to the quality control steps by the UKB, we had to apply additional exclusion criteria to the UK Biobank participants, in line with standard practice in genome-wide association studies (Winkler et al. 2014) (Supplementary Figure S1). As such, we further excluded individuals with documented and inferred sex chromosome abnormalities and those that had a low call rate (less than 99.0%) or excessive heterozygosity as determined by the UKB. Finally, we restricted the analyses to European ancestry (determined by the genotype principal components) as well as unrelated individuals, resulting in a total analytical dataset of 295,150 samples (Table 1). Further quality control measures based on the genetic dosage/intensity estimation are indicated below. The current study was conducted as part of the registered project 22,224. The UK Biobank obtained broad informed consent from its participants and has been approved by IRBs at participating sites. Access to the data implies that the proposed analyses are in agreement with the relevant IRB approvals.

### Variables evaluated

The age and BMI of the individuals were recorded at the time of blood draw and smoking status were ascertained at baseline interview via questionnaire. The frailty index (FI) consisting of 49 health-related items, was computed as recently described (Williams et al. 2019) for all participants from questionnaire data. Briefly, the Rockwood accumulation deficit model was applied summarizing the number of self-reported diseases, symptoms and signs as well as overall health assessments in each individual. The FI ranges from zero to one and represents the fraction of the actual number of health deficits reported by an individual divided by the total number of potential health deficits considered (i.e. queried in the questionnaire). Increased frailty of an individual represents a major risk factor for future diseases as well as mortality. Recently, the UKB measured a panel of 29 biomarkers in urine and blood and the data were released to the public after extensive quality control. In addition, platelet, red and white blood cell counts were measured as an absolute number per unit volume and the abundance of individual blood cell classes such as lymphocytes, monocytes, neutrophils, eosinophils, basophils were recorded in percent (%) of white blood cells. Calibration and quality control were performed by the UK Biobank investigators. All-cause mortality and thus survival time after recruitment was ascertained from the date and cause of death reported up until 2018-02-11 for all deceased UK Biobank participants (mean follow-up time 8.90 years).

### Computation of mitochondrial abundance in UK Biobank participants

In the UKB participants, we determined somatic mitochondrial DNA abundance [as an approximation of the number of mtDNA copies (Longchamps et al. 2020)] from the intensities of genotyping probes on the MT chromosome. On Affymetrix arrays, the relative amount of DNA hybridized to the array at each probe is expressed as the log2 ratio (L2R), which represents the log2 transformed ratio of the observed genotyping probe intensity divided by the intensity at the same probe observed in a set of reference samples.

Initially, we computed the median L2R values across all 265 variants passing quality control on the MT chromosome with MoChA (MOsaic Chromosomal Alterations caller) (Loh et al. 2020). Similarly, we also computed the median L2R value across 12 probes considered to be probes which are highly predictive of mtDNA abundance on the Affymetrix 6.0 Genome-wide SNP array. This approach is equivalent to the approach used in the MitoPipeline implemented in Genvisis (Tin et al. 2016), however only 12 out of the 25 high-quality probes necessary for the MitoPipeline algorithm are present in the Axiom chips (correlation between both estimates *R* = 0.90).

To compare those array-based estimates to another method of mtDNA abundance determination, we also computed the average coverage of the MT genome normalized to the total genomic coverage as estimated with mosdepth (Pedersen and Quinlan 2018) in around 50,000 individuals with whole-exome sequencing (WES) data in the UK Biobank (Van Hout et al. 2019). Since no capture probes for mtDNA sequences were used for the WES enrichment process, this method quantifies the off-target reads that map to the MT genome. Prior studies indicated that this approach yields accurate mtDNA abundance estimates that are highly correlated to qPCR estimates of mtDNA abundance (Zhang et al. 2017; Picardi and Pesole 2012; D’Erchia et al. 2015). However, we noticed that normalized MT coverage was negatively correlated to the mtDNA abundance values ascertained with MoChA (*R* = − 0.07) or with the MitoPipeline approach (*R* = − 0.06). This finding indicated that several poorly performing probes were confounding the abundance estimation.

We, therefore, fit a multivariate linear regression model to select intensities that are statistically significantly predicting the normalized MT coverage from exome sequencing data (Supplementary Table S1). We then multiplied the respective L2R value of each probe by the weight in Supplementary Table S1 and computed the median L2R value across those weighted L2R values (mL2RMT), resulting in a single mtDNA abundance estimate for each individual. The distribution was rescaled to have a mean of zero and a standard deviation (SD) of one within each genotyping plate consisting of 96 samples. The weighted mtDNA abundance estimate was positively correlated to the normalized mean mitochondrial exome sequencing coverage with a spearman rank correlation coefficient of 0.33 (Supplementary Fig. 1), indicating that they should capture the same biological signal, i.e. mtDNA abundance. To assess this and to compare our method of mtDNA abundance quantification to the (unweighted) approaches, we computed the association of different mtDNA abundance estimates with those established variables (Supplementary Fig. 5) such as age (McArdle et al. 2013; Moore et al. 2018), sex (Knez et al. 2016), white blood cell count (Shim et al. 2020), smoking (Cardellach et al. 2003) as well as BMI (McArdle et al. 2013). We found that the estimates from the weighted computation of mtDNA abundance as well as the normalized MT coverage accurately reflected mtDNA abundance in agreement with prior reports (Supplementary Fig. 5). Importantly, the effect sizes large agreed between those approaches, indicating that they capture the same biological signal that is mtDNA abundance. On the other hand, the unweighted approaches yielded contradictory results and therefore do not agree with prior findings by other groups, suggesting that they are a poor predictor of mtDNA abundance. To further analyse potentially haplogroup specific effects, haplogroups in the UKB participants were estimated with *haplogrep2* (version 2.1.25) (Weissensteiner et al. 2016) from all MT variants genotyped on the chip with standard settings. We only analysed the top-level haplogroups that were present in more than 1000 individuals due to power considerations.

### Additional quality control based on the intensity and whole-exome sequencing data in the UKB

Similar to the quality control steps suggested for Illumina genotyping arrays, for each individual, we used MoChA to compute the standard deviation of the L2R values (SDL2R) of all autosomal variants and only included individuals with SDL2R of less than 0.36 (i.e. less than two SDs from the mean SDL2R of all samples), effectively excluding samples with excessive variation in their genotyping intensities. Furthermore, we excluded individuals with an b-allele frequency phase concordance across phased heterozygous sites greater than 0.52 (Vattathil and Scheet 2013). In addition, we also computed the average autosomal DNA abundance from all autosomal L2R values (mL2Rauto) with MoChA. Adjusting for this variable allows us to account for differences in genotyping quality and differences in DNA hybridization to the genotyping chip. Furthermore, we compared the association results from the mtDNA abundance to the overall (autosomal) DNA abundance to identify MT specific effects.

### Statistical analyses

We coded the continuous mtDNA abundance (mL2RMT) as the outcome and used linear regression in *R* (function lm as implemented in base *R*) to evaluate its correlation with modifiable (life-style) and non-modifiable exposure variables. In the PheWAS, the mtDNA abundance distribution was split into tertiles (cut-offs: − 0.43 and 0.39 SD from the mean) and considered the exposure for the diseases (i.e. outcome). All analyses were adjusted for age at baseline/blood draw, sex (determined from the genotyping data), the first two genotype principal components (PCs), the number of missing genotypes as well as genotyping batch. We also performed some association testing by stratifying patients according to their MT haplogroup. Those analyses were additionally adjusted by the quality (i.e. confidence) of the respective haplotype computation as reported by *haplogrep2*. To determine whether mtDNA abundance is correlated to age in a U-shaped fashion (i.e. differently between pre- and post-menopausal women), we computed a two-lines test. This approach fits two regression models for women above and below a programmatically determined age threshold, respectively, as proposed by Simonsohn (2018). Unless otherwise mentioned, to account for multiple testing (where appropriate), we controlled the false-discovery rate to be less than 5% and thus report results that have a *Q* value of less than 0.05.

### Genome-wide association study of mitochondrial abundance

The genome-wide association study (GWAS) of mitochondrial DNA abundance was computed with plink2 (Chang et al. 2015). In general, rare mutations are harder to call and usually have more missing genotypes. Since mtDNA abundance is associated with missingness, investigating rare variants might result in many false-positive findings, which are reflective of high missingness. We, therefore, restricted the analyses to variants with a minor allele frequency greater than 5% as well as those with less than 2% missing data, a MACH *R*^2^ imputation quality greater than 0.6 as well as not deviating significantly (*P* < 10^–05^) from Hardy–Weinberg equilibrium.

The association between mtDNA abundance and the additive genotypes in the genome-wide association study was adjusted for by the first 10 genotype PCs, age at baseline, sex, genotyping batch, genotyping missingness/call rate as well as neutrophil percentage, lymphocyte percentage and white blood cell count. The genomic inflation factor *λ* was calculated with the *GenABEL* library version 1.8-0 implemented in *R*. We considered variants associated with mtDNA abundance with a *P* value lower than 5 × 10^–08^ to be genome-wide significantly correlated to mtDNA abundance. Functional mapping of associated variants to genes within genome-wide significant loci was done with FUMA (Watanabe et al. 2017). Independent signals within each locus were defined as additional association signals at genome-wide significance and with a correlation (*R*^2^) of less than 0.1 to the variant with the lowest *P* value. In each locus, we considered the variant (as well as correlated variants at *R*^2^ > 0.6) within gene bodies with the highest CADD (Rentzsch et al. 2019) score to be the most likely functional variant and also recorded the affected gene (i.e. labelled each locus according to the gene name). Similarly, for variants outside of the gene body, we extracted the variant with the lowest RegulomeDB (Boyle et al. 2012) score as well as the closest gene to that variant.

We used fastBAT implemented in the Complex-Traits Genetics Virtual Lab (Cuellar-Partida et al. 2019) using the GWAS summary statistics to assess the genetic burden of variants associated with mtDNA abundance within the gene body of all genes. The statistical significance threshold was set at *P* value < 2.0 × 10^−6^ which is the Bonferroni correction threshold for 20,000 protein-coding genes. Genes with a statistically significant burden were analysed in a pathway overrepresentation analysis (ORA) with Webgestalt as implemented in WebGestaltR (Liao et al. 2019) in R. Pathways for the ORA were retrieved from MSigDB (Liberzon et al. 2011) version 7.1 and we report pathways with a size of between 25 and 1000 genes and with a *Q* value of less than 0.05.

### Heritability estimation of mitochondrial DNA abundance

The phenotypic variance explained by additive contributions from SNPs (SNP heritability) of mtDNA abundance was estimated with the genomic restricted maximum likelihood estimation method implemented in the genome-wide complex trait analysis in BOLT-LMM (Loh et al. 2018), with standard settings and adjusted for the same covariates as described for the genome-wide association analysis.

### PheWAS analysis

Diseases were extracted from the hospital episode spell data of all individuals passing QC and the ICD10 codes were mapped to phecodes (Wu et al. 2019) using the *createPhewasTable* function from the *PheWAS* package (Carroll et al. 2014) implemented in R. This approach combines and maps ICD codes from electronic health records to clinically relevant outcomes, which were manually defined by clinical experts. The hierarchical approach also defines related diseases which are built-in exclusion criteria to prevent contamination of control individuals with cases that have related diseases (Wu et al. 2019). We only considered incident diseases, i.e. diseases that occurred at least once after blood draw/recruitment. We also excluded all patients with prevalent diseases from the respective incident data set so only individuals without pre-existing conditions were evaluated within each respective phecode or phecode grouping which consists of related diseases (Wei et al. 2017). In each association analysis, the controls were individuals that did not have the respective disease or a related disease at baseline nor did they develop the diseases over the follow-up period. The PheWAS was performed with the *phewas* function and we only considered phecodes which occurred in more than 250 cases. In order to reduce the impact of extreme mL2R values and to enable comparison with prior studies (Lan et al. 2008; Kim et al. 2015), we discretized the variable according to tertiles and used this variable as the exposure in the PheWAS. The analyses were adjusted for the same covariates as the genome-wide association study. Results were plotted with the *phewasManhattan* function*.*

## Supplementary Information

Below is the link to the electronic supplementary material.

Supplementary file1 (DOCX 1869 KB)

Supplementary file2 (XLSX 13 KB)

Supplementary file3 (XLSX 10 KB)

Supplementary file4 (XLSX 12 KB)

Supplementary file5 (XLSX 19 KB)

Supplementary file6 (XLSX 69 KB)

Supplementary file7 (XLSX 14 KB)

Supplementary file8 (XLSX 10 KB)

## Data Availability

The data were exclusively retrieved from the UK Biobank and can be accessed upon request from the UK Biobank. The mitochondrial DNA abundance as computed in this manuscript will be reported back to the UK Biobank upon publication. The scripts to compute the weights and the weighted mtDNA abundance in the UKB dataset will be published at https://github.com/GrassmannLab/MT_UKB.
