# Correction to: Deciphering the genetic and epidemiological landscape of mitochondrial DNA abundance

**DOI:** 10.1007/s00439-021-02258-3

**Published:** 2021-02-05

**Authors:** Sara Hägg, Juulia Jylhävä, Yunzhang Wang, Kamila Czene, Felix Grassmann

**Affiliations:** 1 Department of Medical Epidemiology and Biostatistics, Karolinska Institutet, Nobels väg 12A, 171 65 Stockholm, Sweden; 2 Institute of Medical Sciences, University of Aberdeen, Aberdeen, UK

## Correction to: Human Genetics 10.1007/s00439-020-02249-w

In the original article, the acknowledgement section is incorrectly published. The correct acknowledgement is

The computations and data handling were enabled by resources provided by the Swedish National Infrastructure for Computing (SNIC) at UPPMAX, Uppsala University, partially funded by the Swedish Research Council through grant agreement no. 2018–05973.

This research has been conducted using the UK Biobank Resource under Application Number 22224.

