# Supplementary material for: Deciphering the genetic and epidemiological landscape of mitochondrial DNA abundance
Source: Hum Genet. 2020 Dec 31;140(6):849–61. doi: 10.1007/s00439-020-02249-w (PMC8099832; doi:10.1007/s00439-020-02249-w)
Supplement: Supplementary file 1 — Supplementary file1 (DOCX 1869 KB) [file 439_2020_2249_MOESM1_ESM.docx]

**Supplementary Figure S1. Exclusion criteria and quality control in the UK Biobank. A.** Exclusion criteria in the UK Biobank according to established and intensity/dosage specific quality control (QC) criteria. **B.** Distribution of mtDNA abundance as expressed by the median L2R (mL2RMT) of all MT probes across the whole cohort in standard deviations (S.D.) from the mean, coloured by genotyping batch. **C.** To show the reliability of our approach, we have estimated the correlation between mtDNA abundance computed from the weighted genotyping chip intensities and the average coverage of the MT genome (normalized by the total read depth of each individual) in around 50,000 individuals with exome sequencing ^58^. The correlation coefficient is in line with prior studies comparing chip based intensities to exome sequencing data in the Multi-Ethnic Study of Atherosclerosis (MESA, R=0.393 in Longchamps et al. 2020) ^28^. L2R: log2 transformed ratio of the observed genotyping probe intensity divided by the intensity at the probe observed in a set of reference samples; BAF = B-allele frequency


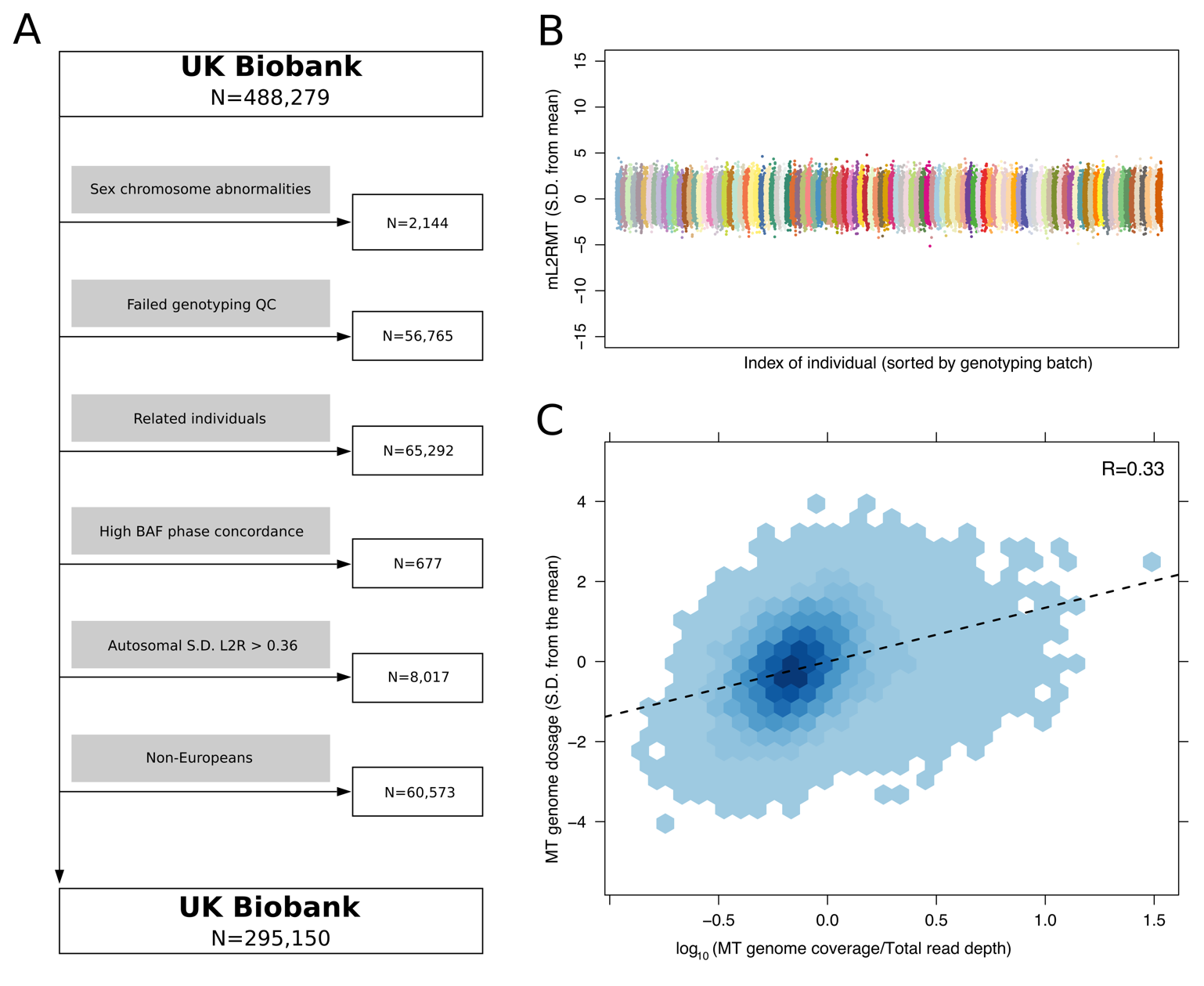


**Supplementary Figure S2. Association of mtDNA abundance with age in males and females.** The distribution of the weighted median L2R values across probes on the MT genome (mL2RMT) by age group and sex. We fit linear regression models separately for women and men below and above 55 years of age, respectively. A significant positive correlation between age and mtDNA abundance was observed in pre- and peri-menopausal women. In contrast, mtDNA abundance decreased in post-menopausal women as well as across all age groups in men. The number of individuals within each age group are depicted in the respective bar.


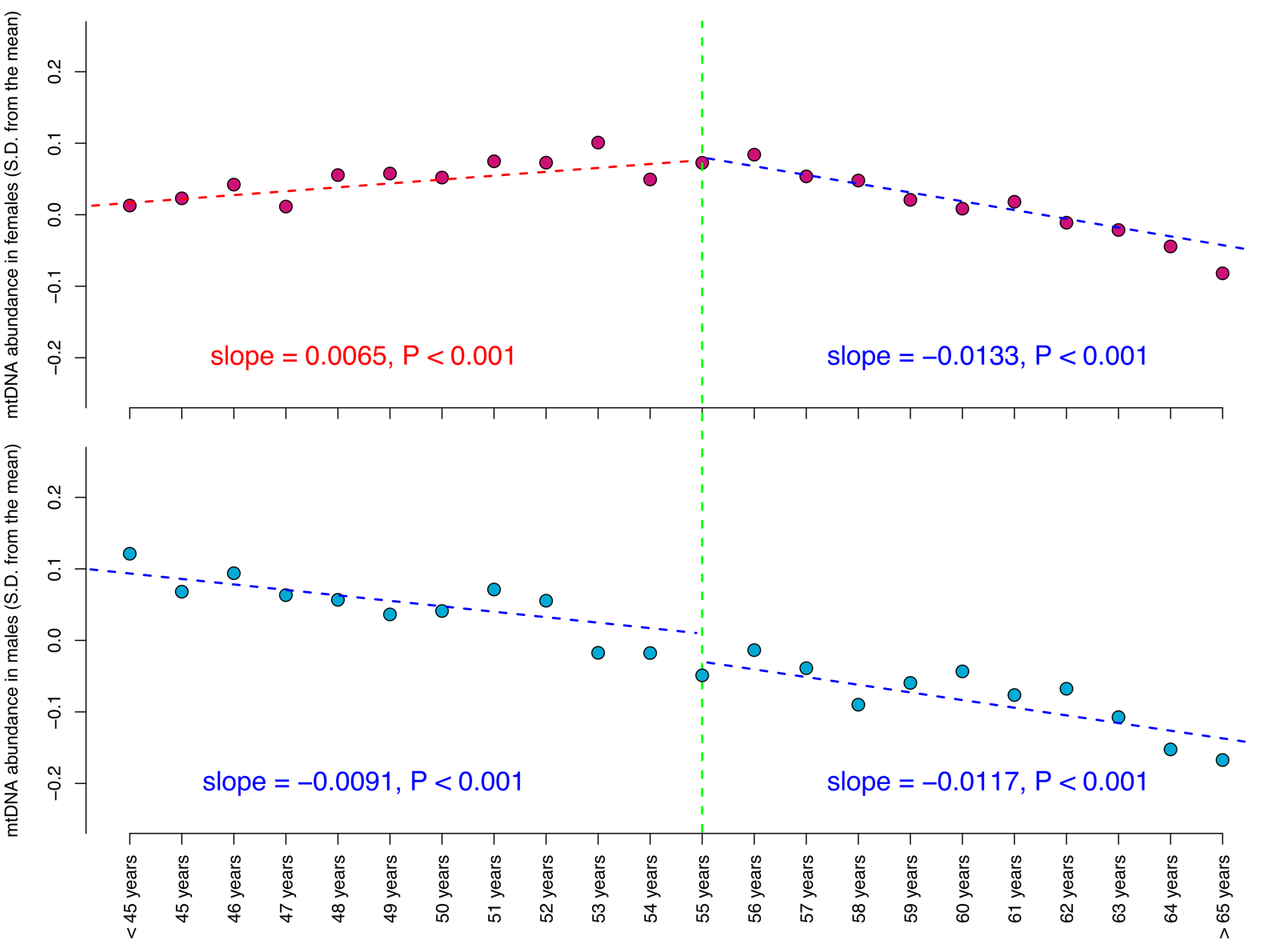


**Supplementary Figure S3. Distribution of mtDNA abundance in common haplogroups within the UK Biobank.** The distribution of the weighted median L2R values across probes on the MT genome (mL2RMT) by major haplogroup. Only haplogroups with at least 1,000 individuals are plotted. The haplogroups all have significantly different mtDNA abundance estimates (compared to haplogroup H, which is the most common haplogroup).*** P-Value < 0.001. 95% CI: 95% confidence intervals; S.D. standard deviation


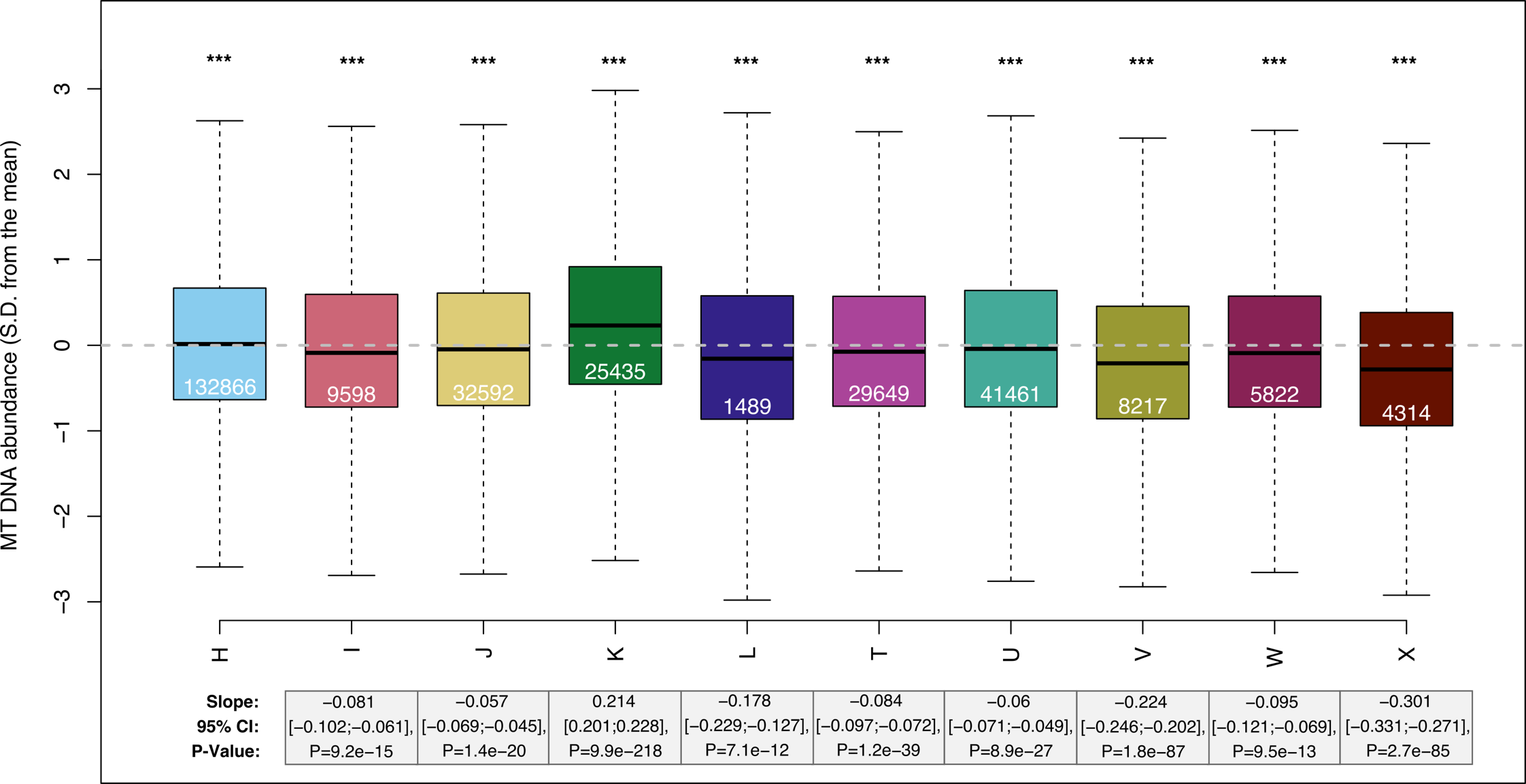


**Supplementary Figure S4. Pathway overrepresentation of genes with a significant burden of common variants associated with mtDNA abundance.** A pathway enrichment analyses was performed with WebGestaltR to estimate the overrepresentation of genes with a significant burden due to common mtDNA abundance associated variants. Pathways statistically significantly enriched (Q-Value<0.05) are highlighted with solid circles, those below statistical significance are indicated with a transparent grey circle. Selected descriptions for statistically significant pathways are shown.

**
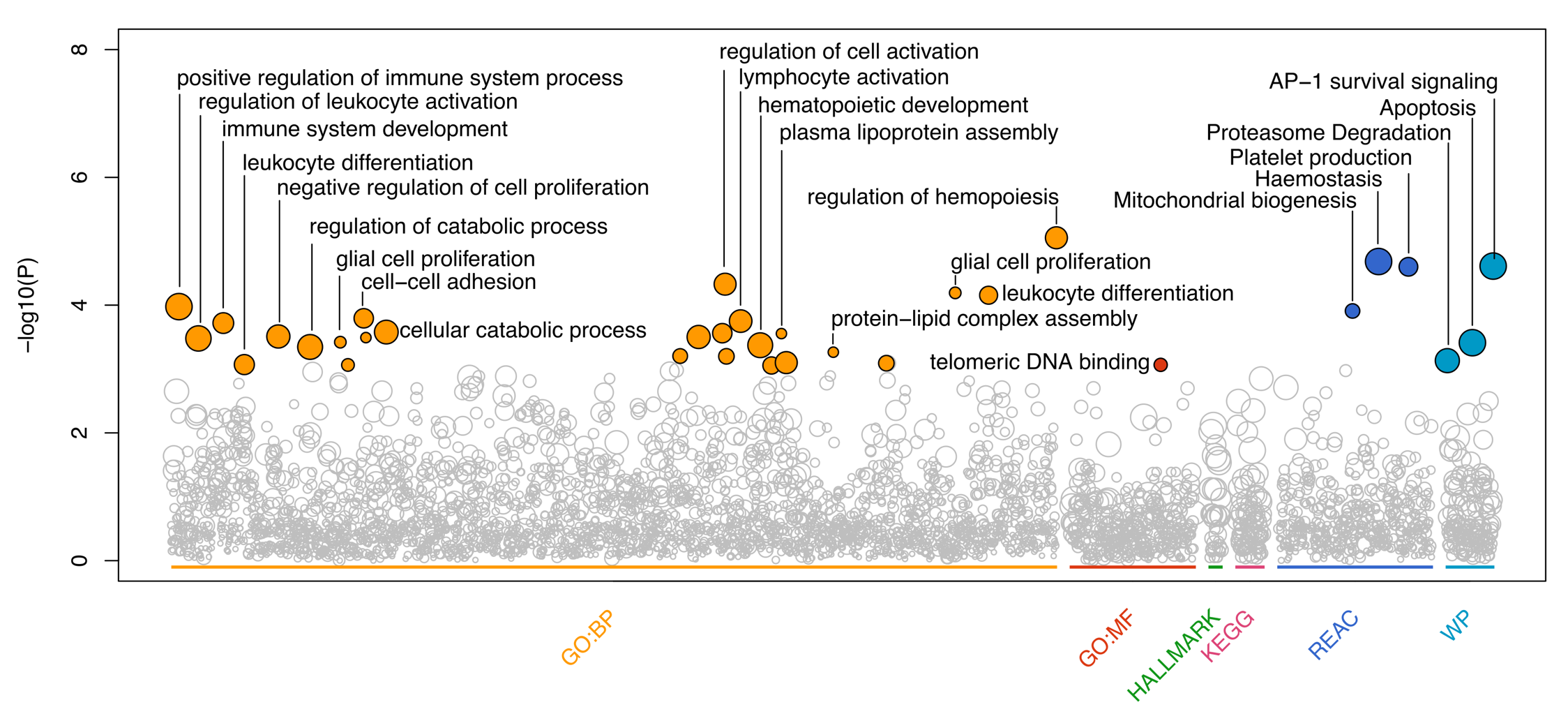
**

**Supplementary Figure S5. Association of different mtDNA abundance estimates with factors previously reported to influence mtDNA abundance. A.** The size and colour (see colour bar) of the circle represent the slope of the association of five features with different mitochondrial abundance estimates (mL2RMT): (1) The intensities of 45 high quality probes were weighted (**Supplementary Table S1**) to more accurately capture the mtDNA abundance as estimated from the whole exome sequencing (WES) reads. From the weighted intensities, we computed the median L2R in all UKB participants. (2) The MT genome coverage computed from the off-target reads obtained in WES was normalized to the total read depth in around 50,000 individuals. (3) The unweighted mL2RMT (MoChA) is computed from the intensities of all 265 probes passing QC on the Axiom genotyping chip mapping to the MT genome. (4) Similarly, the unweighted mL2RMT (MitoPipeline) estimate is computed from 12 high quality probes on the MT genome and thus represents an approach similar to the MitoPipeline, as implemented in the Genvisis program. **B.** The numerical effect sizes, 95% confidence intervals and P-Value of the associations presented in A. The weighted mtDNA abundance (mL2RMT) based on 45 probes showed a similar pattern of association as the normalized MT genome coverage estimated from the off-target reads obtained in whole exome sequencing. In contrast, estimating mtDNA abundance from the unweighted probe intensities resulted in associations that were not in agreement with the literature such as increased mtDNA abundance with advanced age. Correlations which were statistically significant (Q-Value<0.05) are indicated with an asterisk. Nominally significant associations (P-Value<0.05) are shown as circles with black borders. Packyears, number of years smoking one pack of cigarettes a day; BMI, body mass index; mL2RMT, median log 2 ratio of mitochondrial probes;

**
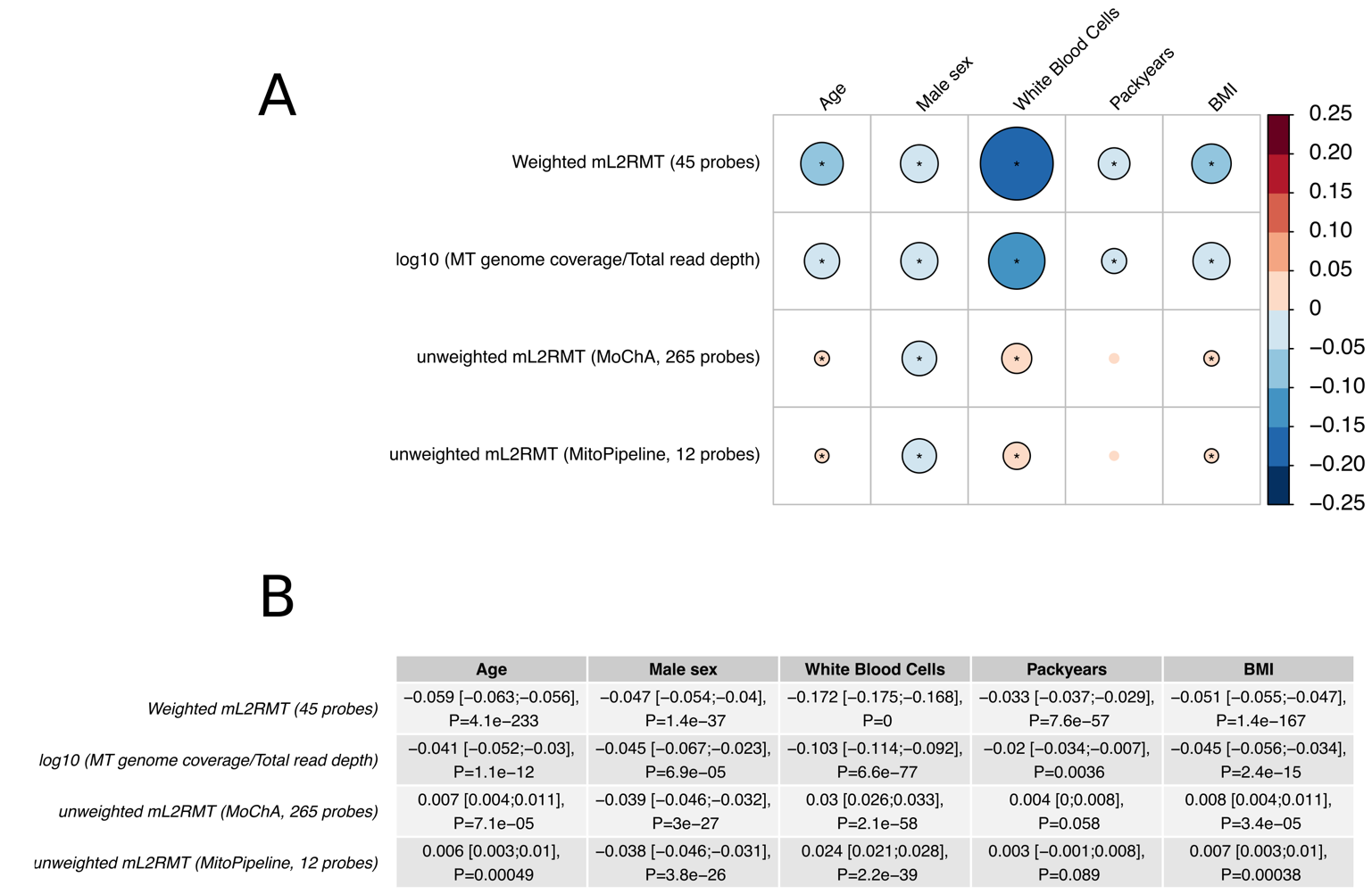
**

**Supplementary Table S1. Weights used to compute the mtDNA abundance from genotyping probe intensities (L2R)**

| **Chromosome** | **Probe** | **Position [hg19]** | **Allele 1** | **Allele 2** | **Weight** |
| --- | --- | --- | --- | --- | --- |
| MT | Affx-34461939 | 235 | A | G | -0.064 |
| MT | Affx-89025736 | 1391 | T | C | 0.128 |
| MT | Affx-89025696 | 1406 | T | C | 0.067 |
| MT | Affx-34461788 | 1438 | A | G | 0.069 |
| MT | Affx-79381658 | 1719 | G | A | 0.019 |
| MT | Affx-89025695 | 2218 | C | T | 0.040 |
| MT | Affx-34461959 | 2706 | A | G | 0.150 |
| MT | Affx-34461963 | 2758 | G | A | 0.087 |
| MT | Affx-89025737 | 3308 | T | G | -0.063 |
| MT | Affx-89025676 | 3423 | T | G | -0.046 |
| MT | Affx-79443419 | 3645 | T | C | 0.119 |
| MT | Affx-79381662 | 3666 | G | A | -0.052 |
| MT | Affx-89025682 | 3866 | T | C | 0.091 |
| MT | Affx-89025729 | 3949 | T | C | -0.056 |
| MT | Affx-79381667 | 3992 | C | T | -0.047 |
| MT | Affx-89025770 | 4093 | A | G | -0.042 |
| MT | Affx-89025668 | 4171 | C | A | 0.062 |
| MT | Affx-34462075 | 4769 | A | G | 0.028 |
| MT | Affx-34462083 | 4883 | C | T | 0.056 |
| MT | Affx-79381672 | 5004 | T | C | -0.090 |
| MT | Affx-79381674 | 5442 | T | C | 0.029 |
| MT | Affx-34462180 | 6752 | A | G | -0.010 |
| MT | Affx-34462190 | 7028 | C | T | 0.054 |
| MT | Affx-79443447 | 7476 | C | T | 0.137 |
| MT | Affx-34462282 | 8869 | A | G | -0.123 |
| MT | Affx-89025719 | 8993 | T | G | -0.057 |
| MT | Affx-89025749 | 8994 | G | A | -0.032 |
| MT | Affx-34462338 | 9716 | T | C | -0.071 |
| MT | Affx-79381690 | 10398 | A | G | 0.034 |
| MT | Affx-79381691 | 10463 | T | C | 0.050 |
| MT | Affx-34461600 | 10688 | G | A | 0.065 |
| MT | Affx-89025768 | 11025 | T | C | 0.048 |
| MT | Affx-34461623 | 11251 | A | G | -0.038 |
| MT | Affx-89025753 | 11299 | T | C | -0.034 |
| MT | Affx-89025715 | 11674 | C | T | -0.029 |
| MT | Affx-34461750 | 13789 | T | C | 0.057 |
| MT | Affx-89025700 | 13879 | T | C | 0.098 |
| MT | Affx-89025746 | 14167 | C | T | -0.051 |
| MT | Affx-86496743 | 14318 | T | C | -0.125 |
| MT | Affx-89025726 | 15250 | C | T | -0.055 |
| MT | Affx-34461828 | 15452 | C | A | 0.071 |
| MT | Affx-89025690 | 15693 | T | C | -0.103 |
| MT | Affx-89025698 | 15812 | G | A | 0.076 |
| MT | Affx-79381714 | 15833 | C | T | -0.081 |
| MT | Affx-79443532 | 16362 | T | C | 0.041 |

**Supplementary Table S2. Effect sizes (slope), 95% CIs and P-Value of associations depicted in Figure 1**

**Supplementary Table S3. Effect sizes (slope), 95% CIs and P-Value of associations depicted in Figure 2A**

**Supplementary Table S4. Effect sizes (slope), 95% CIs and P-Value of associations depicted in Figure 2B**

**Supplementary Table S5. Genome-wide significant loci associated with MT abundance**

**Supplementary Table S6. Genes with a significant burden of common variants**

**Supplementary Table S7. Pathway overrepresentation analysis (ORA) results**

**Supplementary Table S8. Phenome-wide significant diseases associated with MT abundance**
